# Cerebral organoids as a new model for prion disease

**DOI:** 10.1371/journal.ppat.1009747

**Published:** 2021-07-21

**Authors:** Bradley R. Groveman, Anna Smith, Katie Williams, Cathryn L. Haigh

**Affiliations:** Prion Cell Biology Unit, Laboratory of Persistent Viral Diseases, Rocky Mountain Laboratories, National Institute of Allergy and Infectious Diseases, National Institutes of Health, Hamilton, Montana, United States of America; University of Calgary, CANADA

The rapid development of induced pluripotent stem cell (iPSC) technology has considerably expanded the range of human cell and tissue models available for investigators. IPSCs can be used to generate a variety of cells and tissues, with examples ranging from directed differentiation of cell populations for studying homogenous cultures to more complex three-dimensional (3D) structures. One especially advantageous innovation was the development of mini-tissues termed “organoids.” Organoids allow cells to be studied in a 3D environment that loosely mimics how they would interact with neighboring cells in an organism. This is critical because cells inside an organ are structured as part of their function, something that is rarely achievable when cells are cultured in vitro. Human brain has proven difficult to model, and this tissue is not widely available for study. Therefore, the application of organoid technology to produce 3D cultures of self-patterning brain tissue, human cerebral organoids (hCOs), offers great promise for investigating how brain cells become dysfunctional and die in neurodegenerative conditions.

Prions diseases (PrDs), or transmissible spongiform encephalopathies, encompass a family of aggressive neurodegenerative conditions that affect both humans and animals. These diseases are caused by misfolding of a cellular protein, the prion protein (PrP), into misfolded conformers (prions or PrP^Sc^) that can then template conversion of more misfolded PrP^Sc^. This templated misfolding results in transmissibility of PrDs within a species and, under certain circumstances, across species. Certain hallmark features are common to most PrDs, including deposition of PrP^Sc^ in the brain, vacuolation giving the brain a spongy appearance, and astrogliosis. In human PrDs, different subtypes of disease have different clinical presentations and different biochemistry. The subtypes can be modeled in mice but only recently, with the development of new iPSC-based models, has it been possible to consider the pathogenesis of human PrDs in a fully human cell system. Below and within Fig 1, we outline current progress and future possibilities of hCO systems for the investigation of PrDs.

**Fig 1:**
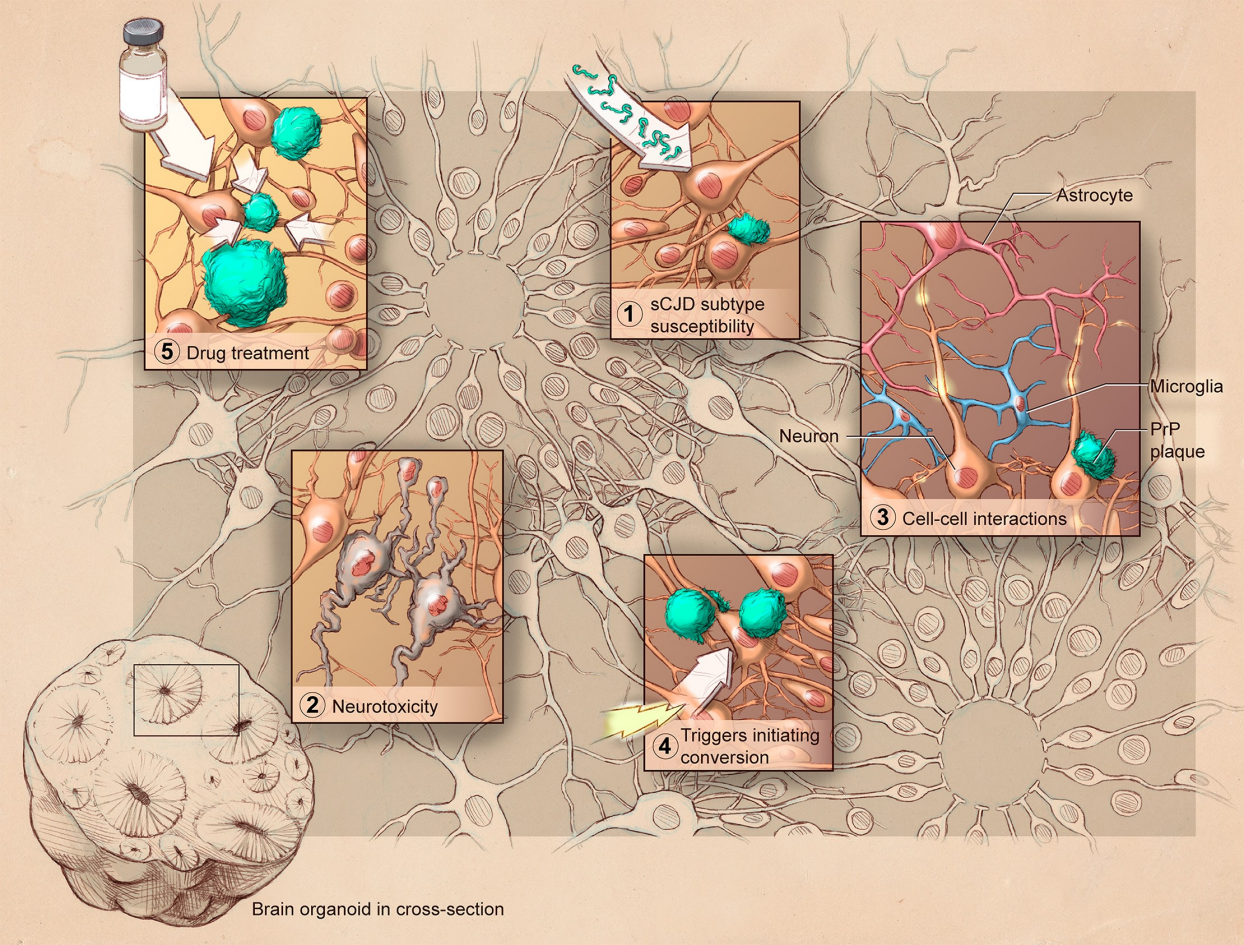
Graphic representation of the use of hCOs to study prion diseases. Organoids permit investigation into various aspects of prion disease including (1) different sCJD subtypes; (2) neuronal dysfunction and death; (3) cellular cross-talk; (4) conversion initiating triggers; and (5) efficacy of putative therapeutics. hCO, human cerebral organoid; PrP, prion protein; sCJD, sporadic Creutzfeldt–Jakob disease.

## Organoids become infected with and accumulate human prions

Until 2017, no reproducible human cell model of prion infection existed. There was precedent in the literature from mouse models that neural stem cells (NSCs), and mature cultures differentiated from NSCs, would be permissible to infection [1–3]. Thus, iPSCs were a prime technology to apply to this problem. The first system derived from human iPSC lines for PrD research studied astrocytes [4]. Transmission from human brain tissue to cultures of iPSC-derived astrocytes demonstrated that it was feasible to transmit prions from human tissue to human tissue cultures and that the biochemical features of the subtypes were preserved [4]. Using hCO cultures differentiated from iPSCs employing the method described by Lancaster and Knoblich [5], our group was able to confirm that infection could be transmitted to organoids [6]. The biochemical presentation of infection was influenced both by the host (infected) cells and by the infecting inoculum, indicating that the organoids may be able to discern biological characteristics of the different infecting subtypes.

## Organoids can model neuronal dysfunction and neuronal death

Modeling neuronal dysfunction and death in cell culture models of PrD has traditionally been challenging as cells often show little or no damage when infected. Being able to investigate disease phenotypes in hCOs offers enormous benefit. From the initial infections of the hCOs, there were hints that dysfunction was occurring. Over the course of infection, changes in cellular metabolism were seen, and a shift in cytokine secretion began to occur from about 90 days postinfection [6]. Cytokine detection showed a similar increased in chitinase 3–like-1 secretion as seen in human brain tissue taken from patients who died of prion disease [7]. This parallel with human disease, coupled with the previous demonstration of cell death in hCOs used to model other infectious brain diseases [8], demonstrates that organoids have potential to uncover pathways causing neuronal dysfunction and death during PrDs.

## Organoids permit study of cell–cell interactions

Understanding the failure of neurotransmission during PrD is of clear importance, and neuroelectrophysiology approaches have been developed to measure hCO synaptic transmission [9]. Neurons are not the only cells critical for neurotransmission and neuroplasticity; astrocytes and oligodendrocytes are both intricately involved. Organoids develop astrocytes and oligodendrocytes from about 2 months to 5 months old [10]. Astrocytes influence establishment of prion infection and disease course [11], and the hCO model permits in vitro investigation of the cross-talk between astrocytes and neurons. Other cells are also involved in maintaining brain health, including microglia and endothelial cells, but these do not originate from the neuroectoderm lineage used to differentiate the neural lineage cells. Therefore, different approaches are being developed to study these cells.

Microglia, as the immune cell of the brain, play a critical role in the homeostasis of healthy brains [12,13]. Microglial involvement in PrDs has been somewhat controversial, with animal studies showing both neuroprotection and potential late stage neurotoxicity [12,13]. Recent advancements in hCO technology have been made by the introduction of microglia-like cells into mature hCOs [14] and by protocols innately developing microglia within hCOs [15]. Abud and colleagues show that iPSC derived microglia-like cells can migrate into hCOs, functionally respond to stimuli, and assume different morphologies [14]. Additionally, Ormel and colleagues demonstrated that microglia can innately develop in hCOs and that these resemble adult microglia [15]. Organoids containing microglial cells advance the new model with the ability to study microglia–neuron interactions and neuroinflammation in PrDs.

## Organoids allow study of PRNP mutations and cellular triggers of prion conversion

It is believed that interactions between host genetics and cellular environment trigger the conversion of PrP to PrP^Sc^ [16–18]. Our group has previously found that hCOs produced from asymptomatic donors carrying the PrP gene (*PRNP*) E200K mutation do not develop any signs of PrD up to 12 months post-differentiation [16]. A similar lack of phenotype was seen when *PRNP* E200K donor-derived organoids were examined for deposition of PrP^Sc^ or Tau [19]. In comparable cultures generated from a *PRNP* Y218N PrD donor, some pathological features, including increased phosphorylated Tau and cell death, were present in the absence of disease-associated PrP [20]. This suggests that cellular environment triggers could be highly important for conversion and that certain disease-associated changes in cell biochemistry may not require prion propagation but instead be linked with PrP dysfunction.

Potential triggers of conversion are numerous. For example, oxidative stress is inherent in aging cells, and both hereditary and sporadic PrDs typically present later in life [18]. Lipid peroxidation (damage caused by oxidative stress) begins early in the course of PrD, and studies have shown that oxidative stress can destabilize native alpha-helices, leading to misfolding and oligomerization [18]. Other studies have implicated cofactors, including nucleic acids (NAs), glycosaminoglycans (GAGs), and redox metals in PrP conversion. NA and GAG binding are thought to lower the free energy barrier for conversion, and dysregulated binding of these molecules could produce a scaffolding that promotes misfolding [17]. Imbalances in redox metal metabolism also disrupt protein structure [18]. Interestingly, Zhang and colleagues found that the E200K mutation perturbs the electrostatic potential on the surface of PrP, which may contribute to dysregulated binding of cofactors leading to disease [21]. Organoids provide a system where these factors can be manipulated to explore their influence on PrP conversion.

## Infected organoids are responsive to anti-prion compounds

In recent years, organoid models of neurological diseases have attracted interest due to their utility in high-throughput and personalized drug screening (reviewed in [22]). The hCO model for PrD [6] laid the groundwork for a human anti-prion therapeutic drug discovery system. Decades of work have yet to identify an effective treatment for PrDs, in part due to the previous lack of human cell–based models [23]. Promising therapeutic approaches continue to be developed; however, these require testing. Exploiting the infectious nature of prions allows testing of prophylactic treatments for use in asymptomatic carriers of genetically unstable PrP and following accidental exposure to prions such as in a hospital setting or by ingesting contaminated meat. This also allows for the testing of therapeutic treatments that are required for symptomatic patients [24]. In the principal study, pentosan polysulfate, an established anti-prion compound, demonstrated the capacity for hCOs to act as a model for drug screening. Both treatment paradigms demonstrated a reduction in PrD indicators [24], validating the hCO model. The ability to monitor disease state and treatment efficacy cheaply and easily will facilitate the race for effective drug treatments for the whole spectrum of PrDs.

In the context of drug discovery, hCOs offer specific advantages. Since PrDs have multiple subtypes, gathering enough patients with a particular subtype for clinical testing remains a challenge. However, using hCOs, treatment can be tested against any subtype in large numbers. Additionally, iPSCs can be derived from skin samples making patient-specific COs easily obtainable for personalized testing [16]. Furthermore, *PRNP* mutations introduced through CRISPR/Cas-9 technology can generate larger numbers of individual iPSC cell lines than can be obtained from donors with the advantage of isotype controls. The hCO model also provides the unique opportunity to monitor both loss and recovery of neuronal function [25] at the cellular level, which would not be possible in live patients. The capacity for real-time testing in actual human brain tissue improves the chances of finding an effective drug candidate especially when used in combination with traditional animal-based models.

## Conclusions

Cerebral organoids offer an extra dimension to researching human PrD. As is the case for all models, they are not without limitations: they are heterogeneous, do not develop non-neuronal lineage cells, and their size is limited by the diffusion of nutrients to their core. However, new developments are currently under way for advancing hCO culture, such as regional specification, manipulation of cellularity, and 3D-printed vasculature. With the ongoing and rapid development of this technology, it is a promising addition to the available prion models and has great potential to advance the field.
